# Contribution of a Non-β-Cell Source to β-Cell Mass during Pregnancy

**DOI:** 10.1371/journal.pone.0100398

**Published:** 2014-06-18

**Authors:** Chiara Toselli, Colin M. Hyslop, Martha Hughes, David R. Natale, Pere Santamaria, Carol T. L. Huang

**Affiliations:** 1 Department of Pediatrics, Alberta Children’s Hospital Research Institute, Faculty of Medicine, University of Calgary, Calgary, Alberta, Canada; 2 Department of Reproductive Medicine, University of California San Diego, San Diego, California, United States of America; 3 Julia McFarlane Diabetes Research Centre (JMDRC) and Department of Microbiology, Immunology and Infectious Diseases, Snyder Institute for Chronic Diseases, Faculty of Medicine, University of Calgary, Calgary, Alberta, Canada; 4 Institut D’Investigacions Biomediques August Pi i Sunyer, Barcelona, Spain; University of Lille Nord de France, France

## Abstract

β-cell mass in the pancreas increases significantly during pregnancy as an adaptation to maternal insulin resistance. Lineage tracing studies in rodents have presented conflicting evidence on the role of cell duplication in the formation of new β-cells during gestation, while recent human data suggest that new islets are a major contributor to increased β-cell mass in pregnancy. Here, we aim to: 1) determine whether a non-β-cell source contributes to the appearance of new β-cells during pregnancy and 2) investigate whether recapitulation of the embryonic developmental pathway involving high expression of neurogenin 3 (Ngn3) plays a role in the up-regulation of β-cell mass during pregnancy. Using a mouse β-cell lineage-tracing model, which labels insulin-producing β-cells with red fluorescent protein (RFP), we found that the percentage of labeled β-cells dropped from 97% prior to pregnancy to 87% at mid-pregnancy. This suggests contribution of a non-β-cell source to the increase in total β-cell numbers during pregnancy. In addition, we observed a population of hormone-negative, Ngn3-positive cells in islets of both non-pregnant and pregnant mice, and this population dropped from 12% of all islets cells in the non-pregnant mice to 5% by day 8 of pregnancy. Concomitantly, a decrease in expression of Ngn3 and changes in its upstream regulatory network (Sox9 and Hes-1) as well as downstream targets (NeuroD, Nkx2.2, Rfx6 and IA1) were also observed during pregnancy. Our results show that duplication of pre-existing β-cells is not the sole source of new β-cells during pregnancy and that Ngn3 may be involved in this process.

## Introduction

During pregnancy, the maternal pancreas adapts to increased insulin resistance and metabolic demand by up-regulating β-cell mass. A slight β-cell hypertrophy, an increase in insulin synthesis and insulin content, and a lowering of the threshold for glucose-stimulated insulin secretion also constitute part of the β-cell adaptation during pregnancy, which require intact prolactin receptor (PRLR) [1], [2]. Although an increase in β-cell duplication has been consistently observed in pregnancy, the question of whether mechanisms other than proliferation of pre-existing β-cells also contribute to the higher β-cell mass during pregnancy is unclear.

Formation of new β-cells may be accomplished through β-cell replication, differentiation of progenitor/stem cells (β-cell neogenesis), or transdifferentiation (re-programming) from differentiated non-β-cells [3], [4]. Under normal physiological conditions, lineage-tracing experiments confer β-cell proliferation as the main source for new β-cells in the adult pancreas [5], [6]. However, under significant regenerative pressure, β-cell neogenesis and transdifferentiation of other cell types to insulin-producing cells have been reported [7]–[10]. For example, transdifferentiation from alpha- to β-cells has been described in the 99% β-cell ablation model [8], while in the partial pancreatic duct ligation model, recruitment of Ngn3-expressing cells to form mature β-cells has been demonstrated [7], [11]. Furthermore, it has been observed that under certain conditions, cells in the pancreas may recapitulate the embryonic developmental pathway in an attempt to regenerate functional endocrine cells [12]. Whether any of these mechanisms occur under the physiologic stress of pregnancy is still uncertain, as current studies provide conflicting results [6], [13]–[15]. A recent study in humans suggested that formation of new islets, not duplication of β-cells in pre-existing islets, is the main source of β-cell mass increase during pregnancy [13]. This conclusion stemmed from the observation of a higher number of small islets and single β-cells (not associated with the islet) in pancreata of pregnant women compared to non-pregnant women [2], [13], as small islets and single β-cells are often interpreted as evidence of β-cell regeneration. The major caveat here is that lineage tracing in humans is still not available, so the evidence is indirect.

In the current study, we sought to determine whether proliferation of pre-existing β-cells is the only source of new β-cells during pregnancy, using a transgenic mouse that allows lineage tracing of all β-cells. We will also determine whether recapitulation of the embryonic developmental pathways contributes to the β-cell mass expansion of pregnancy by engaging the endocrine fate defining transcription factor Ngn3.

## Materials

### Mice

**Figure 1:**
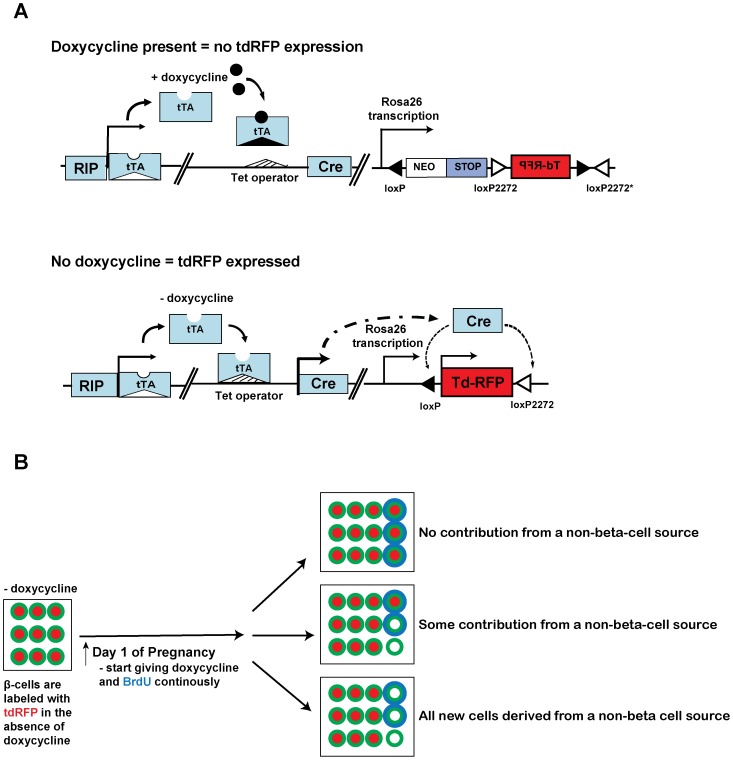
The Cre/loxP-based lineage tracing system in the RIP-Tet-off-tdRFP-positive transgenic mice. **A)** Administration of doxycycline will not allow translocation of Cre and hence, no tdRFP expression. Removal of doxycycline from the RIP-Tet-off-tdRFP-positive mice allows nuclear translocation of Cre, resulting in expression of tdRFP in all insulin-producing cells (RIP =  rat insulin promoter). **B)** A schematic of the sources of new β-cells during pregnancy. On the day when pregnancy was detected (i.e. presence of a vaginal plug in the female), doxycycline is administered to turn off RFP expression. All new β-cells that arose from pre-existing β-cells will be RFP^+^ and insulin^+^ (red filled in green circles). All new β-cells that did not arise from pre-existing β-cells will not express tdRFP (white filled in green circles). Blue outlined circles are BrdU-positive cells.

To be able to genetically (stably) label β-cells with a tracer, we produced mice expressing Cre under the control of a tetracycline-regulatable rat insulin promoter (RIP-tTA/tetO7-Cre, where tTA expressed only in β-cells binds to the tet07 element, leading to Cre expression) (Figure 1A). Briefly, a 3.5 Kb NotI-PvuI fragment from a plasmid carrying a RIP-tTA cassette, and a 3.9 Kb PmeI digested fragment from a plasmid carrying TetO7 cloned upstream of Cre-encoding DNA (TetO7-Cre) were co-injected into fertilized oocytes to produce double-transgenic mice. Mice were screened for inheritance of both transgenes by PCR of tail DNA using the following primers: RIP *forward*: 5′- ATTTGAGGGACGCTGTGGGCTCTT-3′; tTA *reverse*: 5′-ATCTCAATGGCTAAGGCGTC-3′; Cre *forward:* 5′- TTTGCCTGCATTACCGGTCG-3′; Cre(IMR1085) *reverse*: 5′- GTGAAACAGCATTGCTGTCACTT-3′. To determine whether these transgenes were functional, we investigated the presence of β-gal activity in islets of RIP-tTA/tetO7-Cre mice carrying a floxed stop-cassette in front of a LacZ gene knocked into the *Rosa26* locus (to be deleted by Cre). One of 5 lines expressed β-gal activity in most islets. Mice born from mothers treated with doxycycline (DOX, which binds to tTA, inhibiting its ability to induce Cre) did not express β-gal, but did so 2 days after DOX withdrawal. We then backcrossed the transgenes of this line onto non-obese diabetic (NOD) mice expressing a powerful, conditional, non-leaky reporter (tandem-dimer red fluorescent protein –tdRFP) targeted in an inverted orientation into the *Rosa26* locus [16]. Unique features of tdRFP vs. other reporters include its non-toxicity and extraordinary brightness, reliable Cre-mediated activation from an antisense orientation with no leakiness, and absence of gene expression variegation effects. We examined pancreas tissue sections from the resulting NOD.RIP-tTA/tetO7-Cre/tdRFP^loxP^ mice for tdRFP expression. Importantly, >90% of all insulin^+^ cells were RFP^+^ and virtually all glucagon^+^ cells were RFP^–^. Additional studies showed that tdRFP was expressed in Pdx-1^+^ cells but not in somatostatin^+^ or PP^+^ cells, demonstrating that expression of tdRFP in this system is highly penetrant and β-cell-specific. All mice were 6–8 weeks old and not diabetic at the time of the experiments. For lineage tracing experiments, mice were fed with doxycycline-supplemented food, starting on the first day of pregnancy and continuously throughout pregnancy, to suppress Cre expression and RFP labeling.

The day after a vaginal plug is found in the female is considered day 1 of pregnancy.

The Ngn3:EGFP (enhanced green fluorescent protein) transgenic mice were purchased from the Mutant Mouse Regional Resource Center, a national network of breeding and distribution facilities serving as National Institute of Health’s premier repository of spontaneous and induced mutant mouse and cell lines (www.mmrrc.org). These mice had their entire coding region of Ngn3 replaced by enhanced green fluorescent protein (EGFP) [17]. The pups were genotyped as previously described [17]. Mice were maintained on 12-h light, 12-h dark cycle with liberal access to food and water. All mice are 8–12 weeks old at the time of experiments. Intraperitoneal glucose tolerance test (IPGTT) was performed after a 14–16-hour overnight fast. Mice were injected with 2 g/kg body weight of glucose (20% D-glucose solution), followed by blood sampling from tail vein for blood glucose at times 0, 15, 30, 45, 60, and 120 min after the injection, using a glucose meter (FastTake).

### Ethics Statement

This study was carried out in strict accordance with the recommendations in the Canadian Council on Animal Care. The protocol was approved by the Animal Care Committee at the University of Calgary (Permit Number: M10070).

### 5-Bromo-2′-deoxyuridine (BrdU) Labeling

Mice started receiving BrdU in drinking water at a concentration of 0.8 mg/ml when pregnancy was detected and for the duration of pregnancy. The day after a vaginal plug is found in the female is considered day 1 of pregnancy.

### Collection and Fixation of Tissue

Pancreata and intestine were isolated from 8–12 weeks old mice and fixed in 4% paraformaldehyde in PBS overnight at 4°C. (Intestine was used as a positive control for Ngn3 expression in the Ngn3:EGFP mice [17] and for BrdU incorporation in the RIP-tTA/Tet07-Cre::ROSA-tdRFP mice). Fixed tissue was washed in cold PBS and cryoprotected with 30% sucrose for 24 h. Tissue was embedded in O.C.T (Tissue Tek) and stored at –80°C. Tissue were sectioned longitudinally to 7 µm, and every 40th tissue section was stained for insulin to identify β-cells. This provides at least 280 µm distance between sections stained, minimizing the chances of sampling the same islet twice.

### Immunofluorescence

Tissue sections were rinsed in PBS three times, and permeabilized with 0.1% Triton X-100 in PBS for 15 min. Antigen retrieval was achieved by incubation in a citrate buffer solution (pH 6.0) at 37°C for 45 minutes. For BrdU staining, tissue sections were also incubated in 2N hydrochloric acid for 30 min at 37°C. After blocking with 5% goat serum or donkey serum at room temperature for 1 hour, tissues were incubated with primary antibodies overnight at 4°C (mouse anti-Ngn3 at 1∶100 from Developmental studies hybridoma bank at University of Iowa; guinea pig anti-insulin at 1∶600 from Dako; rabbit anti-somatostatin at 1∶100 from Sigma; rat anti-BrdU at 1∶100 from Abcam; guinea pig anti-glucagon at 1∶300 from Linco; rabbit anti-RFP at 1∶100 from Rockland; goat anti-GLUT2 at 1∶100 from Santa Cruz; rabbit anti-EGFP at 1∶800 from Invitrogen; goat anti-rabbit Ki67 at 1∶100 from Abcam; goat anti-Pdx-1 at 1∶5000 (kindly provided by Dr. C. Wright), all diluted in 1% goat or donkey serum in PBS). This was followed by fluorophore-conjugated secondary antibodies for 1 hour at room temperature (Cy3-conjugated donkey anti-guinea pig and Cy3-conjugated goat anti-rabbit; both from Jackson ImmunoResearch Laboratories; Alexa-488-conjugated goat anti-rabbit from Molecular Probes, Eugene; Cy5-conjugated donkey anti-rat from Jackson ImmunoResearch Laboratories; FITC-conjugated goat anti-guinea pig from Jackson ImmunoResearch Laboratories; all diluted in1% goat serum/PBS). Bisbenzimide H 33342 trihydrochloride (1 µg/ml; Sigma) was added to the secondary antibody for nuclear staining. Sections were mounted using DakoCytomation fluorescent mounting medium.

### Islet Morphometry

For each pancreas section, consecutive images of adjacent non-overlapping areas of the entire pancreas section were acquired using a Leica fluorescence microscope or an Olympus FV10000 scanning confocal fluorescence microscope, and captured with a CoolSnap digital camera. Images were analyzed by ImageJ software to measure the insulin-positive area as well as the area of the entire pancreas section (identified by nuclear staining). Representative images included in the manuscript were taken on the Olympus IX81 Fluoview (FV1000) laser scanning confocal microscope. β-cell fraction was calculated by dividing the insulin-positive cell area to total pancreatic tissue area on the entire section [2]. β-cell mass was calculated by multiplying β-cell fraction by pancreas weight [2]. Results represent the average of 6–7 tissue sections per animal from 3–5 animals from each group.

### Islet Isolation

Pancreatic islets were isolated from wild type (Ngn3^+/+^) adult female mice. Pancreas was distended using collagenase P (0.66 mg/ml, 2.5 ml/pancreas; Roche), surgically removed, and then incubated at 37°C for 15 min under agitation. The digested pancreas was passed through a sterile 500-*µ*m filter to remove undigested tissue. Centrifugation on a dextran gradient (1.100/1.085/1.075/1.045 g/ml) allowed islet and exocrine tissue separation. Islets were picked off the 1.085/1.075 and 1.075/1.045 g/ml interface and then washed with HBSS (supplemented with 0.25% fraction V BSA from Invitrogen).

### RNA Isolation and Quantitative RT-PCR

Islets were isolated from mice on days 0, 6, 9, and 15 of pregnancy. Total islet RNA (100–200 islets/mouse) was extracted using the RNeasy Mini Kit (Qiagen) following the manufacturer’s instructions. RNA concentration and integrity were assessed using the ND-1000 Spectrophotometer (NanoDrop). cDNA was synthesized using the Quantitect Reverse Transcription Kit (Qiagen). qPCR reactions were carried out in triplicate with QuantiFast SYBR Green Master Mix (Qiagen) at denaturing temperature of 94°C for 2 minutes, followed by 40 cycles of 94°C for 45 sec, 60°C for 45 sec, 72°C for 45 sec, and followed by 72°C for 2 min. Data were collected using the DNA Engine Opticon2 Continuous Fluorescence Detection System and software (Bio-Rad). The relative amount of RNA was determined by comparison with phosphoglycerate kinase 1 (Pgk1) mRNA as a reference gene. Pgk1 was chosen from a standard reference gene panel using both geNorm and NormFinder algorithm Software (TATAA Biocenter). Primers used were: Pgk1 *forward*: 5′-CTCCGCTTTCATGTAGAGGAAG-3′, reverse: 5′- GACATCTCCTAGTTTGGACAGTG-3′; Ngn3 *forward:* 5′-CAGTCACCCACTTCTGCTTC-3′, *reverse:* 5′-GAGTCGGGAGAACTAGGATG-3′ [7]; Pdx-1 *forward:* 5′-AGGTCACCGCACAATCTTGCT-3′, *reverse:* 5′-CTTTCCCGAATGGAACCGA-3′ [7]; Sox9 *forward:* 5′-AGACTCACATCTCTCCTAATGCT-3′, *reverse:* 5′-ACGTCGGTTTTGGGAGTGG-3′; NeuroD1 *forward:* 5′-CAGCATCAATGGCAACTTCT-3′, *reverse:* 5′-GAAGATTGATCCGTGGCTTT-3′ [18]; Nkx2.2 *forward:* 5′-CGGGCGGAGAAAGGTATGGA-3′, *reverse:* 5′-CCGAGCTGTACTGGGCGTTG-3′; CAII *forward*: 5′- CGCTAGACGGACGACAACTT-3′, *reverse*: 5′- CCTTGTGCCAGTTCTCTGGT-3′; IA1 *forward*: 5′- ACGTTTGTCTCGTGGTTGGA-3′, *reverse*: 5′- TAGGCAGAGCAAACCGCATT-3′; Tle3 (QT00117901), Hes-1 (QT00313537) and Rfx6 (QT00130620) are commercial primers from Qiagen. Data collected from thermocycling was compiled and analyzed for significant changes in gene expression using standard GenEx software (TATAA Biocenter).

### Protein Expression Analysis

Whole cell protein extracts were obtained by disrupting the isolated islets in lysis buffer (2% SDS, 125 mM Tris, pH7, 1 mM DTT in PBS, plus protease inhibitors – COMPLETE Mini Tablets, 50 mM NaF, 10 nM okadaic acid, 1 mM Na_3_VO_4_, and 1 mM PMSF) (200 islets/100 ml), sonicated 3 times for 30 seconds, followed by protein concentration determination using the Bradford methods. Protein was separated by SDS-PAGE (30 µg of islet lysates and 10 µg of P19 cell lysates) and transferred onto polyvinylidene difluoride filters, blocked in 3% BSA at room temperature for 1 hour, then incubation with primary antibody (mouse anti-Ngn3 antibody at 1∶100 in blocking buffer, Developmental Studies Hybridoma Bank, Iowa, USA) at 4°C overnight, followed by a 1 hour incubation with horseradish peroxidase-conjugated secondary antibody (donkey anti-mouse antibody at 1∶5000, Amersham). Protein was visualized by the enhanced chemiluminescence method and scanned within the linear range using ImageJ software. Protein expression level was normalized to that of actin, which was used as the loading control. P19 cell lysate was used as a positive control for Ngn3 expression. P19 is an embryonic carcinoma cell line that has the potential of differentiating into cells of the neuronal lineage [19].

### Apoptosis Assay

Pancreas tissue sections from Ngn3^EGFP/+^ mice on days 0, 4, 6, 8, and 14 of gestation (i.e. G0, G4, G6, G8, and G14) were processed to detect the presence of apoptotic Ngn3-expressing cells by terminal deoxynucleotidyl transferase-mediated dUTP nick- end labeling (TUNEL) staining (DeadEnd Colorimetric TUNEL System; Promega). Pancreatic sections were labeled by biotinylated dUTP using the recombinant terminal deoxynucleotidyl transferase.

### Statistical Analysis

All values are represented as mean ± SEM. Statistical analysis was performed with GraphPad Prism IV software using either a student’s *t*-test to compare between two groups, one-way ANOVA coupled with a Tukey’s post-hoc test to compare between 3 or more groups, or a two-way ANOVA coupled with a Tukey multiple comparisons test to compare within and between groups. The minimum level of statistical significance was set at p<0.05.

## Results

### A Non-β-cell Source Contributes to Increased β-cell Numbers during Pregnancy

**Figure 2:**
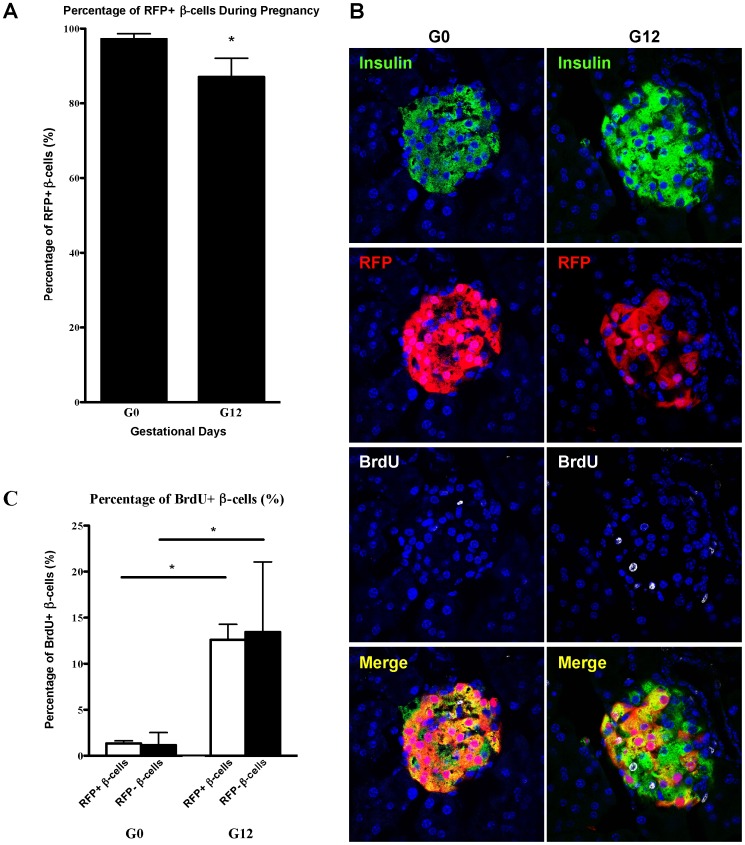
β-cell lineage tracing during pregnancy. **A)** In the non-pregnant RIP-Tet-off-RFP mice, 97.2±1.45% of β-cells express RFP. During pregnancy, only 87.0±5.01% of β-cells express RFP, suggesting a non-β-cells source for the new β-cells. “*”: p<0.05 in comparison to non-pregnant mice by student’s t-test. At least 10,000 cells from 4 separate mice were counted at G0, and the same for G12. **B)** A representative islet from G0 and G12 is shown. Green = insulin, Red = RFP, White = BrdU, Blue = nuclear staining, **C)** Percentage of β-cells that are BrdU^+^. At least 5,000 β-cells from 4 separate mice were counted at G0, and the same for G12. Black bars = RFP^-^ (unlabeled) β-cells. White bars = RFP^+^ β-cells. Comparisons were done by two-way ANOVA with a Tukey multiple comparisons test. “*”: p<0.05 in comparison to G0.

RIP-tTA/Tet07-Cre::ROSA-tdRFP (from herein denoted “Tet-off-tdRFP”) mice were used to heritably mark β-cells and their progeny with RFP. In this mouse, all new β-cells derived from β-cells that were present before pregnancy will be insulin-positive and RFP-positive (insulin^+^/RFP^+^). Starting on the day of pregnancy, the mice were continuously being given doxycycline-containing food for the duration of the pregnancy, and therefore, the Cre is not expressed. As a result, all new β-cells arising from another cell source, i.e. a source other than pre-existing β-cells, will be RFP^-^ and insulin-positive (RFP^−/^insulin^+^) (Figure 1B). Evidence of new β-cells derived from a non-β-cell pool would manifest as a reduction in the proportion of RFP^+^ β-cells. The proportion of RFP^+^ β-cells was calculated as the total number of β-cells that are RFP^+^ relative to the number of β-cells (defined by insulin positivity). When we compared the proportion of RFP^+^ β-cells in the pancreata of non-pregnant (G0) to day 12 pregnant (G12) mice (both received 12 days of doxycycline), we observed a reduction in the proportion of RFP^+^ β-cells from G0 to G12 (97.1±1.45% vs. 87.1±5.01%. p<0.05; >10,000 β-cells from 4 separate mice were sampled at each of G0 and G12) (Figure 2A). This decrease suggests that non-β cells (i.e. unlabeled or RFP^-^) contribute to the formation of new-β-cells during pregnancy. As a control for potential non-specific doxycycline effect, we quantified the proportion of RFP^+^ β-cell in mice that did not receive doxycycline and found similar proportion of RFP^+^ β-cells at G0 and G12 (G0∶99±0.14%, 667 cells counted; G12∶96.4±1.5%, 1199 cells counted).

To determine β-cell proliferation rate, we labeled all dividing cells during pregnancy by providing BrdU in the drinking water for the duration of pregnancy or for a total of 12 days for the non-pregnant mice. We found that 1.30±0.90% of the β-cells are BrdU^+^ at G0, whereas 12.7±3.39% of β-cells are BrdU^+^ at G12. Comparing the RFP^+^ β-cell and RFP^-^ β-cell populations, we found that the proportion of BrdU^+^ cells in the RFP^+^ β-cell population and in the RFP^-^ β-cell population are comparable at both G0 and G12 (G0∶1.32% of the RFP^+^ β-cells are BrdU^+^ and 1.13% of the RFP^-^ β-cells are BrdU^+^; G12∶12.6% of the RFP^+^ β-cells are BrdU^+^, and 13.4% of RFP^-^ β-cells are BrdU^+^) (Figure 2B and C). This suggests that during pregnancy, β-cells derived from both pre-existing β-cells (therefore RFP^+^) and non-β-cell source (therefore RFP^-^) actively divide and contribute to the expanded β-cell mass observed during pregnancy.

### Proportion of Ngn3-expressing Progenitor Cells Decreased during Pregnancy

**Figure 3:**
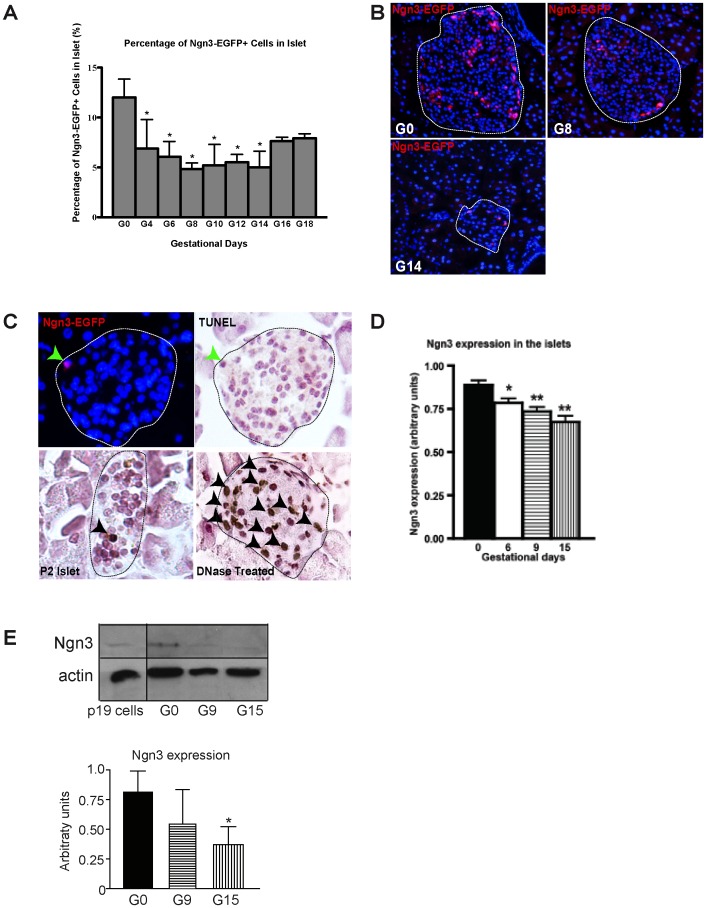
Ngn3-positive cells in pancreatic islets during pregnancy. **A)** Percentage of cells in the pancreatic islets of Ngn3:EGFP mice stained positive for EGFP (therefore expressing Ngn3, these cells will be denoted as Ngn3-EGFP^+^ in all images) but negative for endocrine hormones drops during pregnancy. The number of Ngn3-EGFP^+^ cells was expressed as a percentage of all the cells in the islets. Islets (outlined in white) were identified by staining for endocrine hormones. At least 10,000 cells were counted from each mouse. N = 3–4 separate mice were sampled at each gestational stage. “*”: p<0.05 in comparison to the non-pregnant (G0) mice by student’s t-test. **B)** A representative image of an islet at G0, G8 and G14. **C)** A representative image of TUNEL staining of an islet (outlined) at G6. Islets at postnatal day 2 (P2) and DNase treated pancreas served as controls. Black arrowheads indicate apoptotic (brown) cells. **D)** mRNA levels of Ngn3, as measured by qRT PCR in islets isolated from mice on days 0, 6, 9, and 15 of pregnancy expressed as mean ± SEM; N = 6 separate mice at each gestational stage. All expression levels are normalized to that of PGK-1 (phosphoglycerate kinase 1). “*”: p<0.05 and “**”: p<0.01 in comparison to non-pregnant (G0) mice by one-way ANOVA. **E)** Whole cell lysates were prepared from islets isolated from mice on days 0, 9, and 15 of pregnancy and immunoblotted for Ngn3. The optical density of the Ngn3 band was normalized to that of actin, which was used as the loading control. A representative blot is shown. Results represent mean ± SEM. N = 3–5 mice at each gestational stage from three separate experiments. “*”: p<0.05 in comparison to non-pregnant (G0) mice by one-way ANOVA.

Since all endocrine progenitors transiently expressed high levels of Ngn3 during their ontogeny which then become down-regulated as the progenitors differentiate into hormone producing endocrine cells, we reasoned that a non-β-cell source of new β-cells might involve a population of Ngn3-expressing cells. To determine whether recapitulation of the embryonic developmental pathway by reactivating Ngn3-positive progenitors contributed to the increase in β-cell numbers during pregnancy, we used a transgenic mouse (Ngn3:EGFP mouse) that has previously been used to determine whether β-cell regeneration in injured adult pancreas involves reactivation of Ngn3 [20]. Ngn3 is an early transcription factor that directs progenitors towards the endocrine cell fate. Since EGFP expression is reported to be stable for at least 48 hours, it allows us to detect even a very transient expression of Ngn3 [20]. The presence of EGFP also offers us another marker to detect Ngn3, as Ngn3 protein detection in adult pancreas tissue by immunofluorescence can be difficult. The Ngn3:EGFP mice have one Ngn3 allele replaced by enhanced green fluorescence protein (Ngn3^EGFP/+^). These mice allow recapitulation of spatial expression of Ngn3 in embryonic pancreas (Figure S1A). The heterozygous Ngn3^EGFP/+^ mice have normal glucose tolerance and exhibit a 2-fold increase in β-cell fraction and β-cell mass during pregnancy, comparable to their wild type Ngn3^+/+^ littermates (Figure S1B–E) [2]. When we co-stained the pancreas for insulin and glucagon to identify islets, we found that in non-pregnant mice, approximately 12% of the cells in the islets expressed Ngn3 (identified by EGFP-positivity). This percentage decreased during early pregnancy, nadir at day 8 where only 4.8% of the cells in the islets expressed Ngn3. This reduction in the percentage of Ngn3^+^ cells relative to islet cells remained low throughout pregnancy compared to non-pregnant islets (Figures 3A and B). Interestingly, the percentage of Ngn3^+^ cells relative to islet cells was higher in smaller islets (less than 20 cells) in comparison to larger islets (over 20 cells), in both non-pregnant and pregnant mice (Figure S2A). However, we did not observe a significant difference in the percentage of Ngn3^+^ cells relative to islet cells in small islets between non-pregnant (24%) and pregnant mice (range from 13–20%). Moreover, if an islet was adjacent to the pancreatic duct, we often found the Ngn3^+^ cells in that islet alongside the duct (Figure S2B). We have observed occasional Ngn3^+^ cells on the duct and in the exocrine pancreas, in both non-pregnant and the pregnant mice at similar frequencies (Figure S2C and D).

To determine whether the decrease in the proportion of Ngn3^+^ cells in the islet during pregnancy was due to cell death, TUNEL staining was performed. As we observe the lowest percentage of Ngn3^+^ cells at G8, we would have expected to see the highest proportion of these cells undergoing apoptosis prior to G8. However, we found no apoptotic Ngn3^+^ cells on days 0, 4, 6, 8, or 14 of gestation, after examining at least 50 islets from each of these time points (Figure 3C).

**Figure 4:**
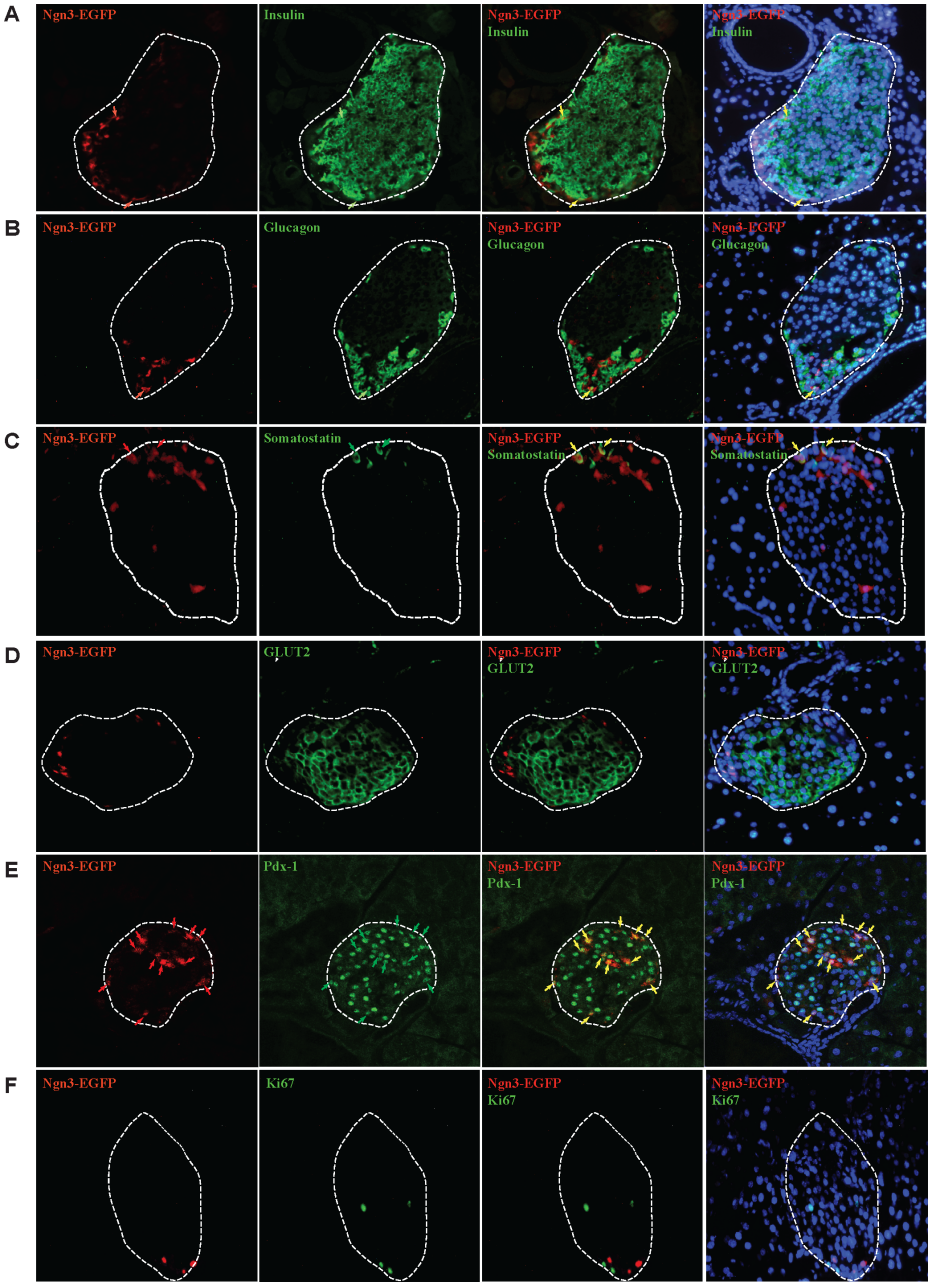
Co-expression of Ngn3 with insulin, glucagon, somatostatin, GLUT2, Ki67, or Pdx-1 in pancreatic islets. Representative images from G8 Ngn3^EGFP/+^ mice are shown immunostained for Ngn3, identified by EGFP-positivity (red), and either insulin (green), glucagon (green), somatostatin (green), GLUT2 (green), Pdx-1 (green), or Ki67 (green) staining. Islets are outlined in white. Red arrows points to Ngn3-EGFP+ cells, green arrows points to hormone-positive cells, yellow arrows points to Ngn3-EGFP+ cells that co-localizes with either a hormone, GLUT2, Pdx-1 or Ki67.

To further characterize the Ngn3^+^ cells, we determined whether they are mitotic (Ki67 positivity), and if they express insulin, glucagon, somatostatin, glucose transporter 2 (GLUT2), and/or Pdx-1 (Figure 4A–F). GLUT2 is a marker of mature β-cells and Pdx-1 is important for β-cell function and insulin transcription [21]. There are small sub-populations of Ngn3^+^ cells that express glucagon and insulin, 6% and 5% respectively, and the proportion of Ngn3^+^ cells co-expressing insulin increased slightly on day 4, 10, and 12 of pregnancy, but not the portion that co-express glucagon (Figure S3 and data not shown). Similarly, a small subpopulation of Ngn3^+^ cells co-expressed somatostatin (Figure 4C). In addition, the majority of Ngn3^+^ cells do not express GLUT2 (Figure 4D), but over 90% of them express Pdx-1 (Figure 4E). The proportion of cells co-expressing Ngn3 and Pdx-1 did not change significantly throughout pregnancy (data not shown). Furthermore, they are non-mitotic as they do not express Ki-67 (Figure 4F).

### Changes in *Ngn3*, *Sox9*, and *Hes-1* Expression

**Figure 5:**
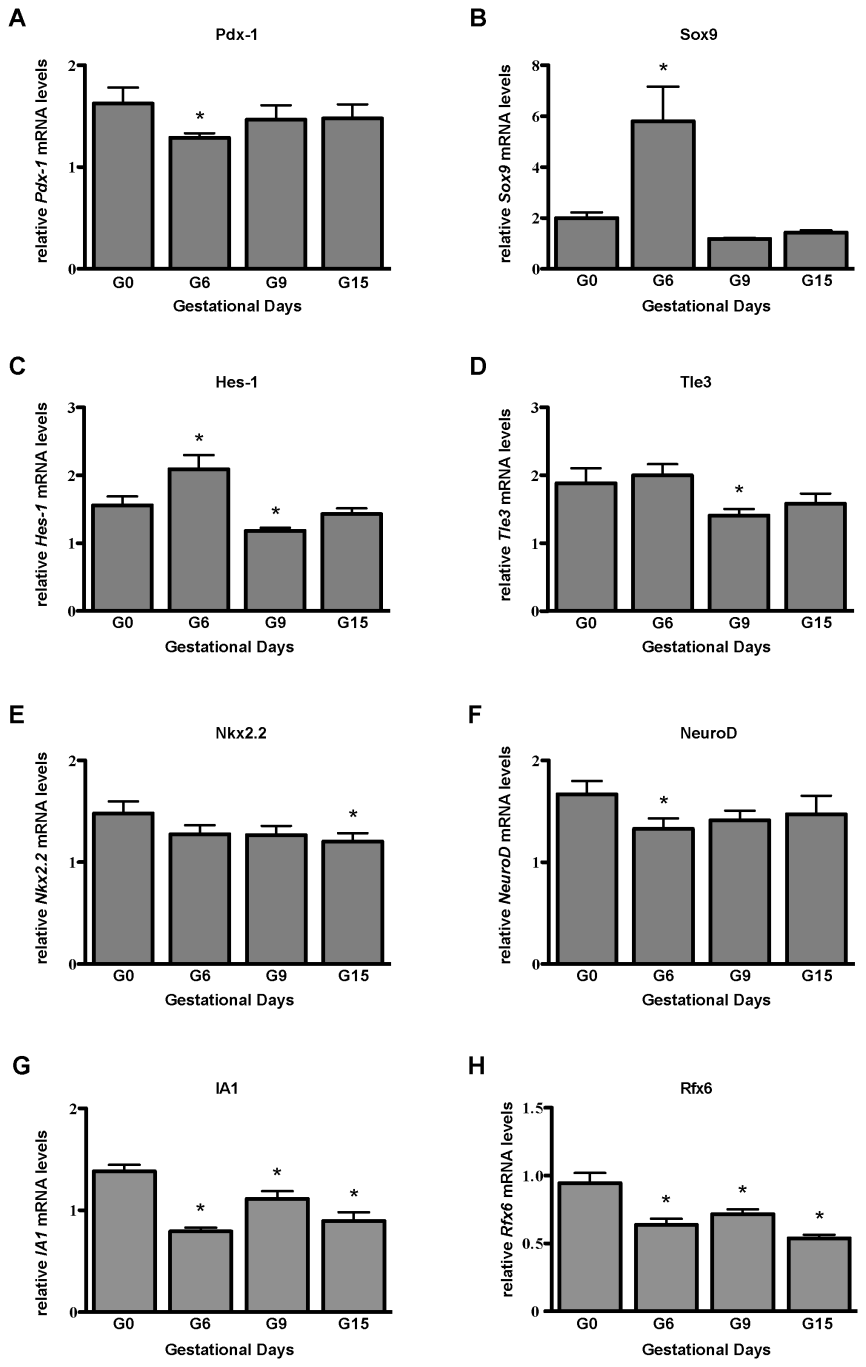
mRNA expression of upstream regulators and downstream targets of Ngn3. mRNA expression of upstream regulators (Pdx-1, Sox9, and Hes-1,) and downstream targets (Tle3, NeuroD, Nkx2.2, IA1, and Rfx6,) of Ngn3. Islets were isolated from Ngn3^+/+^ mice at G0, G6, G9, and G15. Results represent the mean of 3 separate experiments, and expression levels were compared by one-way ANOVA. “*”: p<0.05 by Tukey’s multiple comparison test against G0. N = 6 separate mice at each gestational age.

The reduction in the number of Ngn3^+^ cells prompted us to examine the Ngn3 mRNA expression in the whole islet. Using quantitative real time PCR, we found that *Ngn3* mRNA expression dropped during pregnancy in comparison to day 0 (Figure 3D). A similar reduction in Ngn3 protein expression was also observed (Figure 3E). When we examine expression levels of known upstream regulators of Ngn3, i.e. Hes-1, Sox9, and Pdx-1, as well as downstream targets of Ngn3, i.e. Tle3, Rfx6, IA1, NeuroD and Nkx2.2, we found that *Sox9* and *Hes-1* were up-regulated at G6 in comparison to G0 (Figure 5B–C). Downstream of Ngn3, we found a small but statistically significant reduction in *Tle3, Nkx2.2,* and *NeuroD* expression at G9, G15, and G6 of pregnancy, respectively (Figure 5D–F). Expressions of *Rfx6* and *IA1* in islets also dropped during pregnancy (Figure 5G and H).

## Discussion

The source of new β-cells during pregnancy has not been clearly identified. While some studies have attributed the increase in β-cells numbers during pregnancy solely to β-cell duplication [6], [15], others have postulated that a progenitor cell source may contribute to the increase in β-cell mass during pregnancy [13], [14]. In this study, we aim to determine whether proliferation of pre-existing β-cells is the only source of new β-cells during pregnancy, and whether re-activation of the embryonic pathway involving Ngn3^+^ cells also contributes. Using genetic lineage tracing in adult mice, we found that non-β-cells contribute to the increase in β-cell numbers during pregnancy. Contrary to our hypothesis, the number of Ngn3^+^ cells and the expression of Ngn3 in islets decreased, rather than increased, during pregnancy.

The RIP-tTA/Tet07-Cre::ROSA-tdRFP (Tet-off-tdRFP) mice use a Tet-off system and harbor the tetracycline-controlled transactivator (tTA) gene under the control of rat insulin promoter (RIP). These mice are raised without doxycycline, so insulin^+^ cells expressed RFP. When the mice become pregnant, they are given doxycycline, then RFP expression is turned off, so all new β-cells that are not derived from pre-existing β-cells are RFP^-^ (Figure 1). This is a highly efficient expression system, as 97% of insulin^+^ cells in non-pregnant mice express RFP. However, only 87% of insulin^+^ cells express RFP during pregnancy, suggesting that a small number of insulin^+^ cells came from a source other than pre-existing β-cells. We believe that these RFP^-^ β-cells generated during pregnancy stay in the islets until at least 4 days after the mice give birth, since we found that 86% of β-cells are RFP^+^4 days after the pregnant dams delivers, a proportion similar to that observed on day 12 of pregnancy. We are cognizant of the potential confounding effect of the autoimmune background of the NOD mice on β-cell physiology, but we believe the above result is not merely a strain specific observation or secondary to the autoimmune milieu because: 1) we only used non-diabetic young mice (∼6–8 weeks old), 2) most if not all islets we analyzed had little to no insulitis, and 3) we did not observe any difference in percentage of RFP^+^/Ins^+^ double positive cells between islets that do or do not have insulitis.

Similar to our findings, Abouna et al used a tamoxifen-dependent Cre/loxP-based system that marked β-cells with human placental alkaline phosphatase (HPAP) and found that 44% of β-cells are HPAP-positive before pregnancy which dropped to 33% during pregnancy, suggesting a non-β-cell source of β-cells in pregnancy [14]. Both of these systems, however, do not allow us to identify the origin of this non-β-cell source(s). In a recent study using a nonconditional Cre-mediated mTomato expressing system, Xiao et al searched for evidence of β-cell neogenesis in adult pancreas [15]. In this system, all non- β-cells express mTomato (therefore are red), while all β-cells express GFP (therefore green). Any β-cell that differentiated from a non-β-cell will have a 48-hour window where the cell expresses both mTomato and GFP and therefore appears yellow. When they examined the pancreas on days 14.5–17.5 of pregnancy, no yellow cells were seen, which led to the conclusion that there is no non-β-cell contribution to new β-cells during pregnancy. However, this finding may be reconciled with our observation if earlier stages of pregnancy were examined. Our data suggest that the recruitment of non-β-cells occurs before day 12 of pregnancy, since we found a statistically significant reduction in the percentage of RFP^+^/insulin^+^ cells by day 12, 48 hours earlier than the earliest time point examined by Xiao et al (i.e. day 14.5) [15].

The above observation prompted us to search for a mechanism whereby a non-β-cell source may be recruited to form new β-cells during pregnancy. It has been observed that under certain conditions, cells in the pancreas may dedifferentiate and recapitulate the embryonic developmental pathway in an attempt to regenerate functional endocrine cells [7], [11], [12]. To address this possibility, we set out to determine whether reactivation of the Ngn3 pathway contribute to new β-cells during pregnancy. Ngn3 is a transcription factor necessary for formation of endocrine cells, such that Ngn3-null mice die within the first week of life from diabetes. It is highly expressed in the embryonic pancreas but its expression drops sharply at birth, and its expression level in adult pancreas is less than 1% of that at E15.5 of embryonic development. To our surprise, instead of finding an up-regulation of *Ngn3* expression, as would be predicted if the Ngn3 pathway was activated to recruit more cells to enter the endocrine cell fate, we found a progressive reduction in *Ngn3* expression in the islets during pregnancy. We found that ∼12% of cells in the islet express detectable Ngn3, which dropped to 4.8% by day 8 of pregnancy and the majority of Ngn3^+^ cells did not co-stain for endocrine hormones or the mature endocrine marker, GLUT2, although most expressed Pdx-1. Interestingly, a previous study has reported populations of Pdx-1^+^/insulin-low and Pdx-1^+^/insulin-negative cells in the adult pancreas that had the ability to differentiate into mature β-cells [22]. It is possible that Ngn3^+^ cells in our observations may be comparable to the population described in that study. Whether these Ngn3^+^ cells differentiated into the non-labeled (i.e. RFP^-^) β-cells in our study remains to be determined. Taking advantage of the 24–48 hr expression of EGFP in the Ngn3^EGFP/+^ mice, where EGFP expression is under the control of Ngn3 promoter, we compared the number of EGFP and insulin-double positive cells throughout pregnancy, postulating that if EGFP^+^/insulin^-^ cells differentiate into insulin^+^ cells, we should see an increase in EGFP^+^/insulin^+^ double positive cells during pregnancy. Indeed, we observed a small increase in the percentage of EGFP^+^/insulin^+^ double positive cells during pregnancy (Figure S3). While this small increase in EGFP^+^/insulin^+^ double positive cells during pregnancy may be a result of re-expression of Ngn3, our results suggest that this is not the main mechanism responsible because this increase in EGFP^+^/insulin^+^ double positive cells was observed on days 4, 10, and 12 of pregnancy, at a time when both the number of Ngn3-EGFP^+^ cells (Figure 3A) and the mRNA and protein expression of Ngn3 (Figure 3 D and E) were still dropping in comparison to non-pregnant mice. To directly address this question, it requires labeling all cells that expressed Ngn3 immediately before pregnancy using an inducible Ngn3-Cre transgenic mouse that does not require tamoxifen for gene induction, as tamoxifen has long-lasting negative effects on fertility. Such a transgenic mice is not readily available yet.

Another unanswered question from the present study is the role of transdifferentiation. Transdifferentiation of cells from a mature non-β-cell source to new β-cells has been observed under certain conditions [23]–[25] but not yet reported in pregnancy. Our mouse model does not allow us to investigate this possibility.

The above results do not support reactivation of the Ngn3 pathway as a mechanism of generating more β-cells in pregnancy. Instead, the drop in the number of Ngn3-expressing cells and the down-regulation of *Ngn3* expression in islets during pregnancy is more consistent with the model proposed by Miyatsuka et al, where high levels of *Ngn3* expression was found to keep cells in a state of quiescence while down-regulation of *Ngn3* expression is required for cell proliferation [26], [27]. This suggests that during pregnancy, down-regulation of *Ngn3* in the islets is a prerequisite for β-cell to enter cell cycle and proliferate. To determine what leads to this down-regulation of *Ngn3*, we examined expression of Sox9 and Hes1. During embryonic development, Hes-1 is associated with promoting cell replication and inhibiting cell differentiation and it inhibits expression of Ngn3 [28], [29], whereas Sox9 plays a role in the maintenance of the pancreatic progenitor pool [30] and up-regulates *Ngn3* expression [31], [32]. Here, we found that on day 6 of pregnancy, there was an increase in both *Sox9* and *Hes-1* expression in the islets, a time when the expression of Ngn3 started to drop. Therefore, it appears that the inhibitory effect of *Hes-1* on *Ngn3* expression dominates in this context. Hes1 is a target of NOTCH signaling, which is not usually expressed in adult pancreas. However, in conditions associated with cell dedifferentiation and replication, such as experimental pancreatitis and exposure to cytokines, activation of Hes1 has been reported [33]–[35], and its expression was required for human β-cell proliferation in vitro [36]. Furthermore, Hes1 is also required for maintenance of a progenitor pool, such that Hes1 knockdown has been associated with precocious differentiation and depletion of progenitor pool [37]. Since pregnancy is associated with a drop in Ngn3 expression in the islet, presumably due to the depletion of a β-cell progenitor population that expresses a high level of Ngn3, the transient up-regulation of Hes1 and Sox9 in early pregnancy (i.e. day 6) may serve to maintain a progenitor pool. To confirm that the up-regulation of *Sox9* mRNA expression was not simply due to an increase in its expression in the ductal cells (which express Sox9), we measured expression of carbonic anhydrase II (CAII), which is a marker of ductal cells [38], and found that expression of CAII is stable throughout pregnancy (Figure S4A). We also quantified the Sox9^+^ areas relative to the pancreatic islet area during pregnancy and found that the Sox9^+^ areas did not change during pregnancy (Figure S4B).

When we examined downstream targets of Ngn3, small changes in expression of Tle3 [39], NeuroD, and Nkx2.2 [40] were detected. Tle3 has been shown to promote β-cell differentiation [39]. NeuroD is crucial for pancreas development and it is a transactivator of the insulin gene [41], while Nkx2.2 acts as both a transcription activator for β-cell maturation and function as well as a repressor for alpha- and β-cell formation [42]. Rfx6 regulates islet formation downstream of Ngn3 in both mice and human [43], and IA1 has also been shown to regulate development and differentiation of both pancreatic β-cells and intestinal cells [44]. It is possible that these small reductions in gene expression confer little biological impact, though definitive conclusion requires using gene knockout models that allow titration of gene dosage. Nevertheless, these results are consistent with known functions of genes that regulate β-cell differentiation, and to our knowledge, this is the first study that examined their expression in adult pancreatic islets during pregnancy. These changes in expression of Ngn3 and its regulatory network may hint at genes required for islet adaptations (i.e., β-cell expansion or increased insulin synthesis and secretion) during pregnancy. A clear understanding of the mechanisms engaged during pregnancy to mobilize non-β-cells to take on a mature β-cell phenotype will likely lead to development of therapeutic strategies in conditions of β-cell failure such as diabetes.

## Supporting Information

Figure S1 Characterization of the Ngn3^EGFP/+^ mice during pregnancy. **A)** EGFP expression recapitulates Ngn3 expression pattern in Ngn3^EGFP/+^ mice. A representative image from E15 embryonic pancreas is shown. **B–C)** Intraperitoneal glucose tolerance tests in heterozygous Ngn3^EGFP/+^ mice (EGFP/+) and wild type (+/+) mice on days 15 of pregnancy (G15). Glucose excursions were measured as AUC (millimolar glucose X minutes) and expressed as mean ± SEM; N = 7–14 separate mice for each genotype. **D–E)** β-cell fraction and β-cell mass in non-pregnant (G0) and day 15 pregnant (G15) Ngn3^+/+^ (white bars) and Ngn3^EGFP/+^ mice (black bars) mice. Comparisons between and within a genotype were done by two-way ANOVA with a Tukey’s multiple comparisons test. “*”: p<0.05 in comparison to G0 mice. N = 5–6 separate mice for each genotype at each gestational stage.
(TIF)

Figure S2 Ngn3-expressing cells adjacent to pancreatic duct, on pancreatic duct and in exocrine pancreas. A) Proportion of Ngn3^+^ cells in small islets (less than 20 cells), medium islets (20–99 cells), and large islets (over 100 cells). At least 100 islets were quantified from each mouse. Comparisons between islet size and gestational day were done by two-way ANOVA with a Tukey multiple comparisons test. “*”: p<0.05 in comparison to that in small islets. N = 3–4 separate mice at each gestational day. **B)** Islets adjacent to pancreatic duct with Ngn3-EGFP^+^ cells alongside the duct. **C)** Ngn3-EGFP^+^ cells on duct. A representative image from gestational day 8 is shown. Red arrow indicates Ngn3-EGFP^+^ cell on pancreatic duct. **D)** Ngn3-EGFP^+^ cells in exocrine pancreas. A representative image from gestational day 8 is shown. Red arrow indicates Ngn3^+^ cells in the exocrine pancreas.
(TIF)

Figure S3 Ngn3 and insulin immunoreactivity in β-cells during pregnancy. Percentage of Ngn3^+^ cells co-expressing insulin throughout pregnancy. “*”: p<0.05 in comparison to the non-pregnant (G0) mice. Comparisons were made by one-way ANOVA with a Tukey post-hoc test. At least 500 Ngn3-EGFP^+^ cells were counted at each time point, and >1000 cells were counted at G0. N = 3–4 separate mice at each gestational stage.
(TIF)

Figure S4 Ductal Sox9 expression in islets during pregnancy. **A)** mRNA expression of CAII (marker of ductal cells). Islets were isolated from Ngn3^+/+^ mice at G0, G6, G9, and G15. Expression levels are compared by one-way ANOVA and “*” indicates p<0.05 by Tukey’s multiple comparison test against G0. N = 6 separate mice at each gestational age. No significant differences in *CAII* mRNA expression were observed during pregnancy. **B)** Sox9^+^ area in relation to insulin^+^ (islet) area. No significant differences were detected throughout the gestational period. At least 50 islets were quantified from each mouse. N = 3 separate mice at each gestational stage. **C)** A representative islet (outlined) from G0 is shown. Green = insulin, red = Sox9, blue = nuclear staining, yellow = merge of insulin and Sox9 images. Green arrows indicate Sox9^+^ cells in the islet. White arrowheads indicate Sox9^+^ ducts in the exocrine pancreas. **D)** A representative islet (outlined) from G0 is shown for Ngn3-EGFP^+^ and Sox9 staining. Green = insulin, red = Sox9, white = Ngn3-EGFP, blue = nuclear staining. Yellow arrows indicate Ngn3-EGFP^+^ cell in the islet. White arrowheads indicate Sox9^+^ ducts in the exocrine pancreas. Ngn3^+^ cells were often found adjacent to Sox9^+^ cells.
(TIF)
