# Supplementary figures and images for: Contribution of a Non-β-Cell Source to β-Cell Mass during Pregnancy
Source: PLoS One. 2014 Jun 18;9(6):e100398. doi: 10.1371/journal.pone.0100398 (PMC4062500; doi:10.1371/journal.pone.0100398)

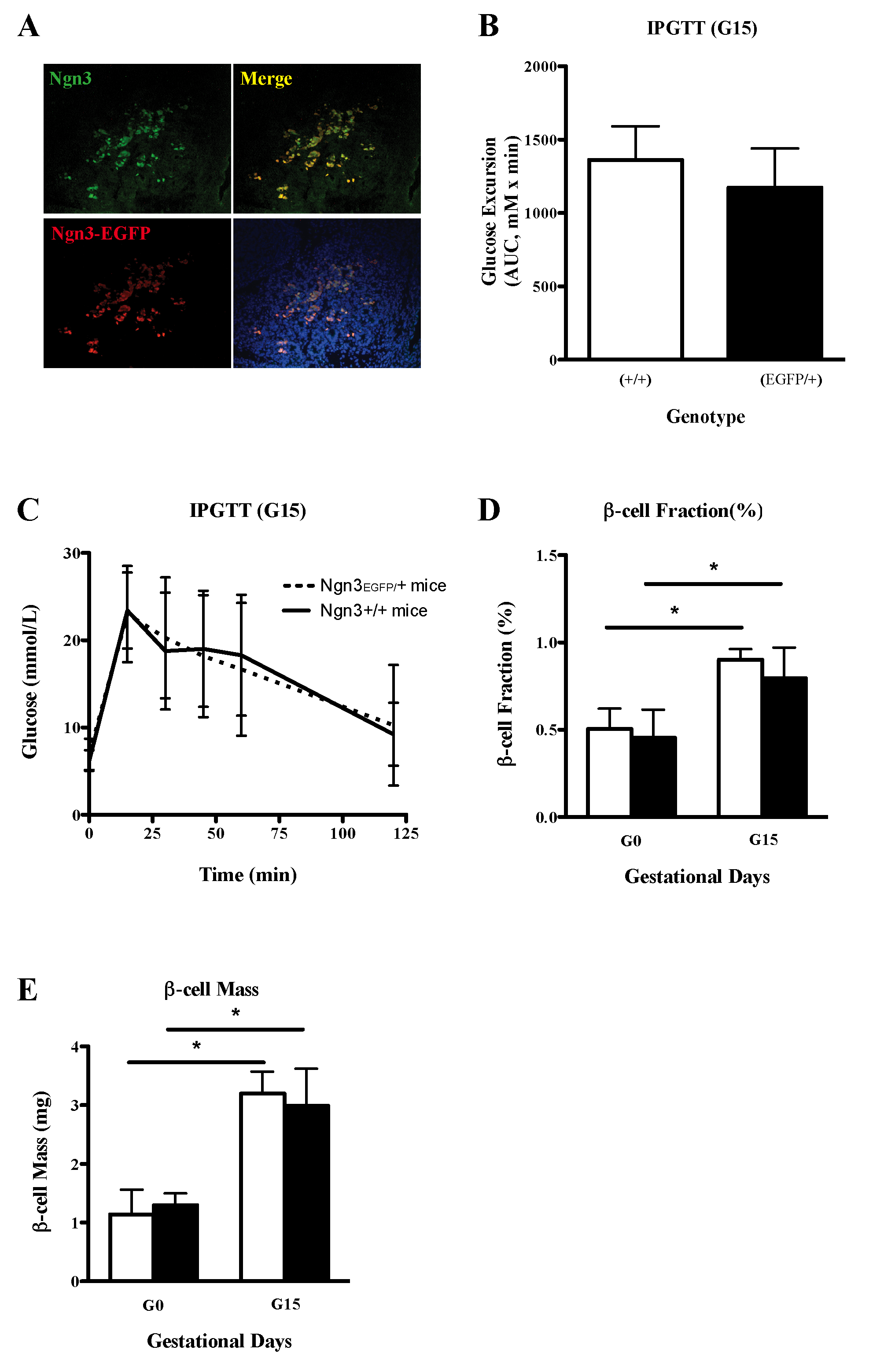

Supplement: Figure S1 — Characterization of the Ngn3EGFP/+ mice during pregnancy. A) EGFP expression recapitulates Ngn3 expression pattern in Ngn3EGFP/+ mice. A representative image from E15 embryonic pancreas is shown. B–C) Intraperitoneal glucose tolerance tests in heterozygous Ngn3EGFP/+ mice (EGFP/+) and wild type (+/+) mice on days 15 of pregnancy (G15). Glucose excursions were measured as AUC (millimolar glucose X minutes) and expressed as mean ± SEM; N = 7–14 separate mice for each genotype. D–E) β-cell fraction and β-cell mass in non-pregnant (G0) and day 15 pregnant (G15) Ngn3+/+ (white bars) and Ngn3EGFP/+ mice (black bars) mice. Comparisons between and within a genotype were done by two-way ANOVA with a Tukey’s multiple comparisons test. “*”: p<0.05 in comparison to G0 mice. N = 5–6 separate mice for each genotype at each gestational stage. (TIF) [file pone.0100398.s001.tif]

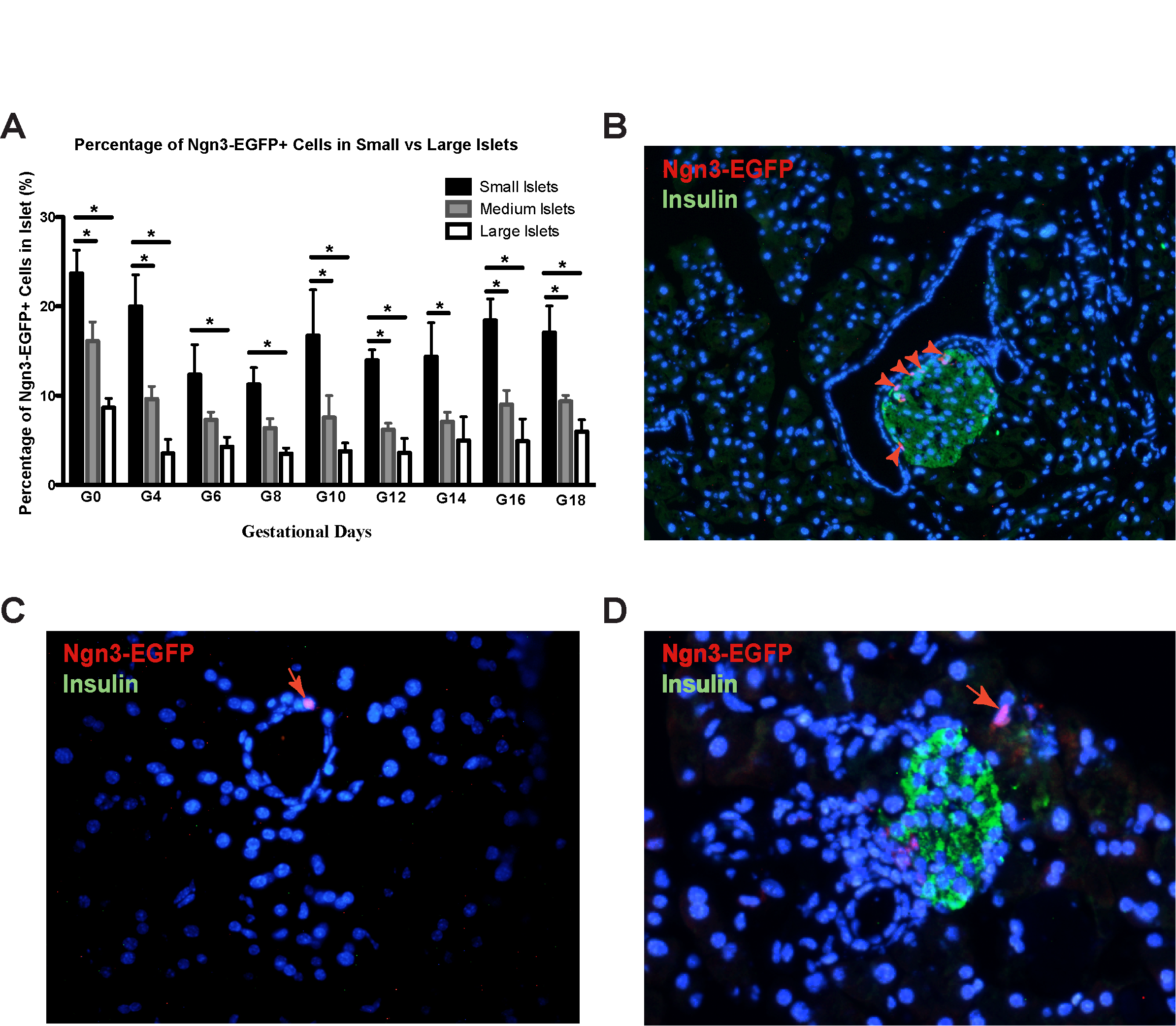

Supplement: Figure S2 — Ngn3-expressing cells adjacent to pancreatic duct, on pancreatic duct and in exocrine pancreas. A) Proportion of Ngn3+ cells in small islets (less than 20 cells), medium islets (20–99 cells), and large islets (over 100 cells). At least 100 islets were quantified from each mouse. Comparisons between islet size and gestational day were done by two-way ANOVA with a Tukey multiple comparisons test. “*”: p<0.05 in comparison to that in small islets. N = 3–4 separate mice at each gestational day. B) Islets adjacent to pancreatic duct with Ngn3-EGFP+ cells alongside the duct. C) Ngn3-EGFP+ cells on duct. A representative image from gestational day 8 is shown. Red arrow indicates Ngn3-EGFP+ cell on pancreatic duct. D) Ngn3-EGFP+ cells in exocrine pancreas. A representative image from gestational day 8 is shown. Red arrow indicates Ngn3+ cells in the exocrine pancreas. (TIF) [file pone.0100398.s002.tif]

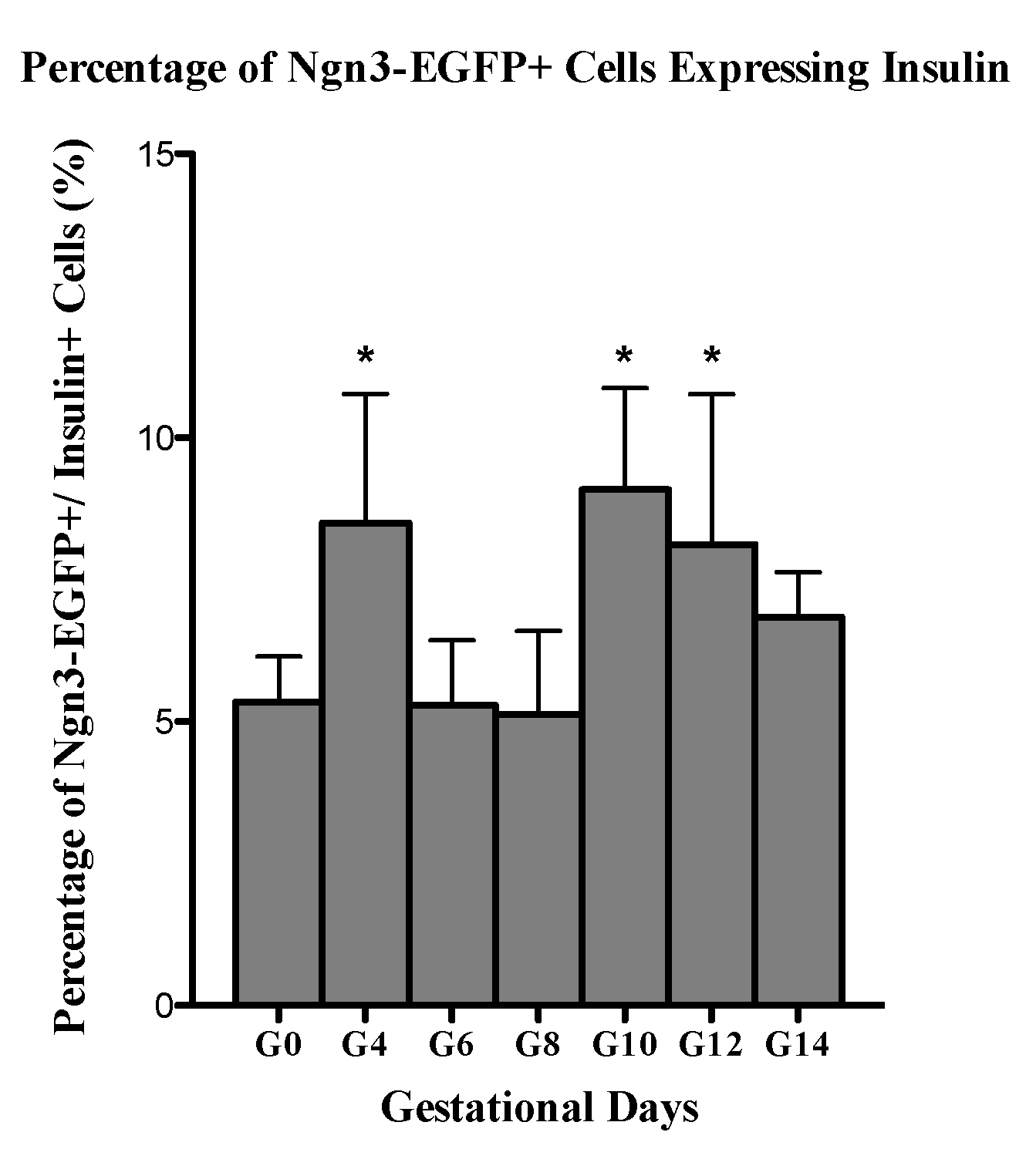

Supplement: Figure S3 — Ngn3 and insulin immunoreactivity in β-cells during pregnancy. Percentage of Ngn3+ cells co-expressing insulin throughout pregnancy. “*”: p<0.05 in comparison to the non-pregnant (G0) mice. Comparisons were made by one-way ANOVA with a Tukey post-hoc test. At least 500 Ngn3-EGFP+ cells were counted at each time point, and >1000 cells were counted at G0. N = 3–4 separate mice at each gestational stage. (TIF) [file pone.0100398.s003.tif]

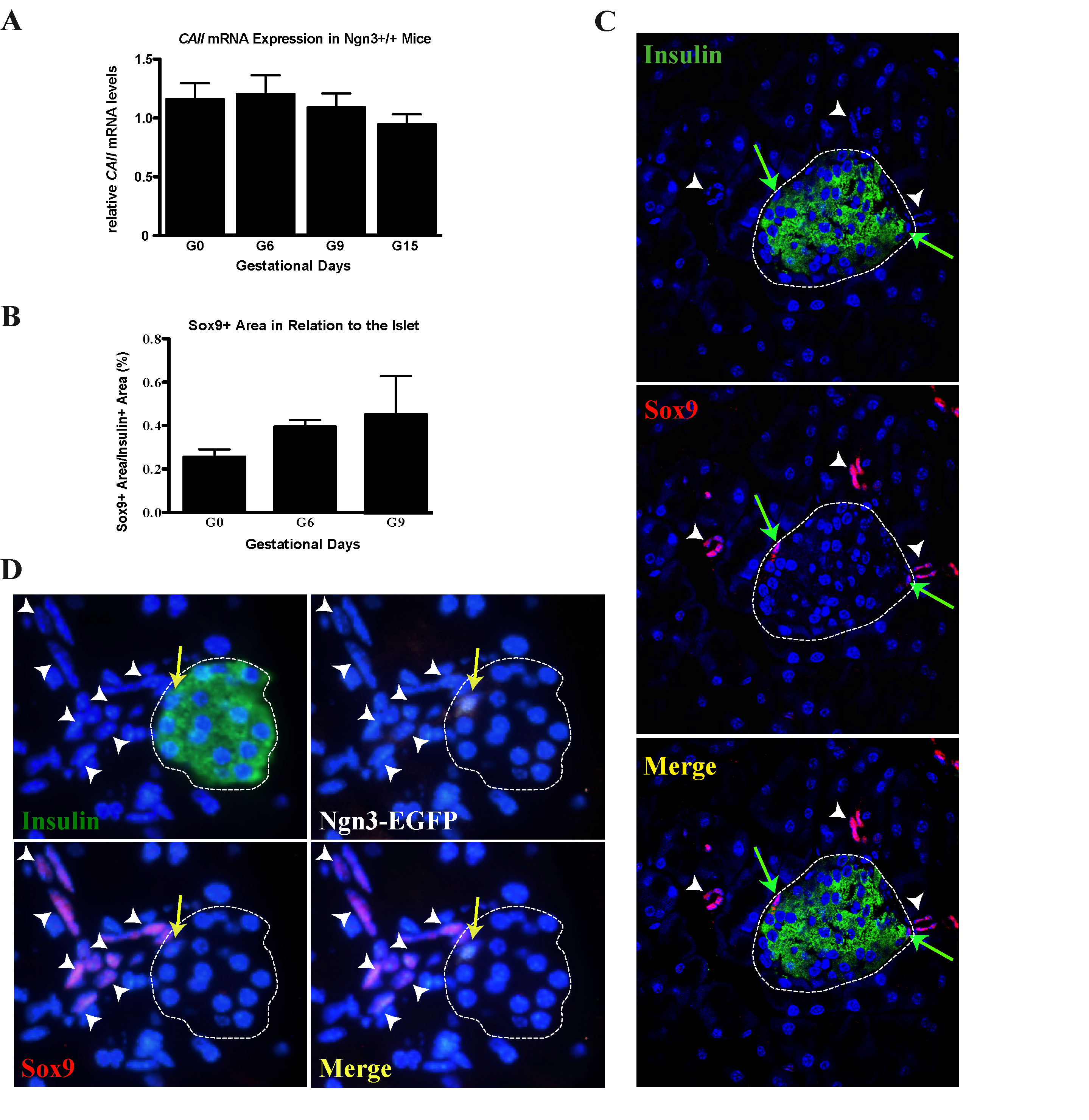

Supplement: Figure S4 — Ductal Sox9 expression in islets during pregnancy. A) mRNA expression of CAII (marker of ductal cells). Islets were isolated from Ngn3+/+ mice at G0, G6, G9, and G15. Expression levels are compared by one-way ANOVA and “*” indicates p<0.05 by Tukey’s multiple comparison test against G0. N = 6 separate mice at each gestational age. No significant differences in CAII mRNA expression were observed during pregnancy. B) Sox9+ area in relation to insulin+ (islet) area. No significant differences were detected throughout the gestational period. At least 50 islets were quantified from each mouse. N = 3 separate mice at each gestational stage. C) A representative islet (outlined) from G0 is shown. Green = insulin, red = Sox9, blue = nuclear staining, yellow = merge of insulin and Sox9 images. Green arrows indicate Sox9+ cells in the islet. White arrowheads indicate Sox9+ ducts in the exocrine pancreas. D) A representative islet (outlined) from G0 is shown for Ngn3-EGFP+ and Sox9 staining. Green = insulin, red = Sox9, white = Ngn3-EGFP, blue = nuclear staining. Yellow arrows indicate Ngn3-EGFP+ cell in the islet. White arrowheads indicate Sox9+ ducts in the exocrine pancreas. Ngn3+ cells were often found adjacent to Sox9+ cells. (TIF) [file pone.0100398.s004.tif]
